# HIV eradication symposium: will the brain be left behind?

**DOI:** 10.1007/s13365-015-0322-6

**Published:** 2015-03-07

**Authors:** B. J. Brew, K. Robertson, E. J. Wright, M. Churchill, S. M. Crowe, L. A. Cysique, S. Deeks, J. V. Garcia, B. Gelman, L. R. Gray, T. Johnson, J. Joseph, D. M. Margolis, J. L. Mankowski, B. Spencer

**Affiliations:** 1 Departments of Neurology, Immunology and Infectious Diseases and Peter Duncan Neurosciences Unit St Vincent’s Centre for Applied Medical Research, St Vincent’s Hospital, Victoria St, Darlinghurst, Sydney, Australia 2010; 2 Department of Neurology, University of North Carolina, Chapel Hill, NC USA; 3 Department of Infectious Diseases, The Alfred Hospital Melbourne Australia, Melbourne, Australia; 4 NeuRA, Sydney, Australia; 5 Burnet Institute, Melbourne, Australia; 6 Department of Infectious Diseases, University of California, San Francisco, CA USA; 7 Division of Infectious Diseases, Center for AIDS Research, University of North Carolina, Chapel Hill, NC USA; 8 University of Texas, Austin, TX USA; 9 Section of Infections of the Nervous System, National Institute of Neurological Disorders and Stroke, National Institute of Health, Bethesda, MD USA; 10 Division of AIDS Research, National Institute of Mental Health, National Institutes of Health, Bethesda, MD USA; 11 Department of Medicine, University of North Carolina-Chapel Hill, Chapel Hill, NC USA; 12 Department of Molecular and Comparative Pathobiology and Department of Neurology, John Hopkins University School of Medicine, Baltimore, MD USA; 13 University of California, San Diego, CA USA

**Keywords:** Human immunodeficiency virus type 1, Acquired immunodeficiency syndrome, HIV-associated neurocognitive disorders, Eradication, Brain

## Abstract

On 18 July 2014, the National Institute of Mental Health in collaboration with ViiV Health Care and Boehringer Ingelheim supported a symposium on HIV eradication and what it meant for the brain. The symposium was an affiliated event to the 20th International AIDS Conference. The meeting was held in Melbourne, Australia, and brought together investigators currently working on HIV eradication together with investigators who are working on the neurological complications of HIV. The purpose of the meeting was to bring the two fields of HIV eradication and HIV neurology together to foster dialogue and cross talk to move the eradication field forward in the context of issues relating to the brain as a potential reservoir of HIV. The outcomes of the symposium were that there was substantive but not definitive evidence for the brain as an HIV reservoir that will provide a challenge to HIV eradication. Secondly, the brain as a clinically significant reservoir for HIV is not necessarily present in all patients. Consequently, there is an urgent need for the development of biomarkers to identify and quantify the HIV reservoir in the brain. Lastly, when designing and developing eradication strategies, it is critical that approaches to target the brain reservoir be included.

## Introduction

On 18 July 2014, the National Institute of Mental Health in collaboration with ViiV Health Care and Boehringer Ingelheim supported a symposium affiliated with the 20th International AIDS Conference. This symposium was directed at HIV eradication in relation to the brain. The purpose of the meeting was to bring the two fields of HIV eradication and HIV neurology together to foster dialogue and cross talk to move the eradication field forward in the context of the brain as a potential reservoir of HIV.

The symposium was divided into six sessions, the first being Eradication: Theory and Practice, the second, Eradication and the Central Nervous System (CNS) Model Systems Including Cells and Animal Models. The third session was devoted to the Brain as a Reservoir and Sanctuary, the fourth was devoted to oral presentations from the attendees. The fifth session identified the Risks and Outcomes of CNS Eradication, and the last was devoted to Current and Future Studies.

## Session 1: eradication: theory and practice

This session provided a theoretical overview of eradication by Dr Margolis followed by a review of whether the brain is a problem for HIV eradication by Dr Deeks.

Dr Margolis began by reflecting on Timothy Brown, the only person known to be cured of HIV infection. Mr Brown was treated for leukemia with two stem cell bone marrow transplants (BMT) and preconditioning that included chemotherapy and total body irradiation (Hutter et al. 2009). Notably, his donor was a CCR5 delta-32 homozygote (Mr Brown was a delta-32 heterozygote). Mr Brown’s clinical course was complicated by graft versus host disease (Hutter et al. 2009). He has been off combination antiretroviral therapy (cART) since 2009 without evidence of viral replication, or latent HIV infection. Dr Margolis reflected on two other HIV positive (HIV+) persons who had ceased cART following successful BMT therapy (the so-called “Boston patients”) and who have remained in HIV remission without detectable plasma RNA or cell-associated DNA for a period of 12 and 32 weeks before experiencing HIV virological rebound (Henrich et al. 2014). He also noted that just prior to the World AIDS 2014 conference, “the Mississippi baby” whose cART had been ceased at 18 months of age, had developed HIV rebound approximately 2 years following cART cessation (Butler et al. 2014).

Dr Margolis then gave “the T-cell centric view” of the first steps towards HIV eradication. Latent HIV infection is present predominantly within central memory resting CD4+ T cells, but also within other T-cell types. When latently infected cells are activated they produce low-level plasma viremia, which is detectable in cART-suppressed individuals using an ultrasensitive single copy RNA assay. Dr Margolis stated that one of the first steps in eradicating HIV infection is to develop an anti-latency strategy to stimulate latently infected T cells to (at least) produce HIV antigen so that the immune system could target and kill these cells (Archin et al. 2014). He noted that over the past 1–2 years strategies involving immunotherapy have being developed (Barouch and Deeks 2014) to complement the anti-latency strategies.

Theoretically, following the administration of the abovementioned treatment strategies in HIV+ patients, Dr Margolis noted that we would still be faced with a number of uncertainties, all of which are fully relevant to the brain, perhaps the most important being the challenge of achieving clearance of all infected cells.

With respect to the CNS, Dr Margolis noted that, as is seen in the plasma of treated HIV+ patients on virologically suppressive cART, very low-level residual viremia is detectable in the cerebrospinal fluid (CSF). He noted that while there is the challenge of achieving adequate antiretroviral drug penetration into the CNS (Letendre et al. 2010), a CNS antiretroviral intensification study using raltegravir failed to reduce low-level CSF viremia (Dahl et al. 2011) similar to other studies undertaken in the periphery (Gandhi et al. 2012; Llibre et al. 2012). Therefore, Dr Margolis stated that he was not sure that finding low levels of HIV RNA in the CSF is very different from finding low-level HIV RNA in the periphery, or other tissues. With respect to this, he observed that the brain does have long-lived tissue macrophages (microglia) that might harbor HIV latently. Notwithstanding, he noted, as did several other speakers on the day, that the hypothesis that the CNS serves as a reservoir site for HIV remains to be conclusively proven.

Were this found to be the case, the issue of achieving effective antiretroviral CNS penetrance would be important and Dr Margolis highlighted some research directed towards providing cART via nanoformulations to enhance their CNS levels (Dou et al. 2009).

The issue of establishing an ideal model to study HIV persistence in different tissues including the brain was discussed (Dinoso et al. 2009). Dr Margolis noted that primates can now be successfully virologically suppressed on cART and in several laboratories, primates who have received suppressive cART for 2–3 years, are being studied to determine the presence of HIV tissue reservoirs including the CNS. Humanized mice models are being used also to study CNS reservoirs (Denton et al. 2012).

Dr Margolis then reviewed some of the latency reversing agents (LRAs) being evaluated to activate latently infected cells. His group has studied the histone deacetylase (HDAC) inhibitor vorinostat and found that it increased the levels of HIV gag RNA in resting CD4+ T cells of eight HIV+ patients (Archin et al. 2012a). Work undertaken by Bullen et al. found that none of several LRAs, including vorinostat, induced viral outgrowth from latent reservoirs of HIV+ patients on suppressive cART (Bullen et al. 2014). Only one highly toxic LRA, bryostatin, caused significant fold increases in HIV mRNA both intracellularly and in the culture supernatant of the patients’ resting CD4+ T cells in one study (Bullen et al. 2014).

Future work will therefore examine the efficacy of combining different LRAs to activate latent HIV cellular reservoirs. Dr Margolis discussed work being done by different pharmaceutical companies including Merck and Gilead to screen compounds for their ability to reverse HIV latency. Dr Hazuda at Merck has been evaluating farnesyltransferase inhibitors (FTIs), which alone, or in combination with vorinostat appear to increase expression of HIV in latent HIV reservoirs. FTIs will be evaluated in animal models in the future.

Dr Margolis then discussed the important question of whether serial treatments with LRAs or combination LRA treatment will be necessary to purge HIV fully from all cellular and tissue reservoirs. This seems likely because it appears that a cascade of molecular events must occur at the HIV promoter for transcription to occur, some of which happen by “chance” or in a “stochastic” manner. Hence, he noted that serial treatments, using different combinations, might be necessary to fully clear HIV reservoirs, akin to the approach undertaken with cancer chemotherapy.

Dr Margolis then referenced the very sobering, recent work undertaken in Robert Siliciano’s laboratory (Ho et al. 2013), which compounds the challenges faced by the HIV cure field. Here, Ho et al. showed that a proportion of the viruses that had previously been thought defective because they could not be induced from patients’ latent CD4+ cell reservoirs following in vitro activation, *are* in fact replication competent (Ho et al. 2013). Ho et al. estimate that the size of the replication-competent HIV reservoir is up to 60-fold greater than had been previously estimated (Ho et al. 2013).

Dr Margolis noted that in various cell populations including those within the brain, some of the drugs’ targets might be different and documenting this will be important if we need to target cells in the brain. For example, macrophages versus T cells have much higher levels of HDAC8 enzymes than the HDAC 1–3 enzymes that predominate in T cells. Hence, the field will need to know whether HDAC inhibitors used in clinical trials will target HDACs in different cell types, including microglia and brain macrophages.

Turning to clinical treatment trials in human subjects, Dr Margolis felt that it will be hard to harvest meaningful CNS samples from patients hence the use of magnetic resonance spectroscopy imaging and gold standard serial neurocognitive testing will be important to elucidate the impact of different treatment strategies upon the CNS.

In closing, Dr Margolis said that although the brain is challenging because of the difficulty in obtaining samples and the brain’s different cell types, that his approach to “attacking” persistent neuro HIV infection is identical to his approach to persistent systemic HIV infection. Studies need to be undertaken in cell lines, primary cell models, patients’ cells, animal models, and patients. He stated that we need to approach the issue of persistent viremia in resting CD4+ T cells and determine which other cells contribute to ongoing low-level viral replication and identify drugs that can extinguish this replication. With respect to persistent provirus in resting CD4+ cells and other possible cell types, he stated that we will continue to study HDAC inhibitors, and other novel anti-latency agents singly and in combination.

Dr Deeks offered “an agnostic perspective” of whether the CNS can serve as an HIV reservoir for HIV+ persons receiving virologically suppressive, long-term cART. He posed the following four questions to provide an overview of his “agnostic perspective”.

### Does HIV reside in the CNS during long-term cART?

Dr Deeks noted that in both humans and SIV primate models, HIV infects the brain during primary HIV infection and during untreated HIV/SIV infection. Dr Deeks referred to the work of Schnell et al. demonstrating that genetically distinct HIV variants exist in the CNS and plasma (Schnell et al. 2011). He also cited Dahl et al. who showed that in patients with HIV-associated dementia (HAD), genetically diverse macrophage-tropic and R5 T-cell-tropic viruses are present in the CSF and evince slow and rapid decay kinetics, respectively following cART commencement (Dahl et al. 2014a). The findings of Dahl et al. suggest that there are long-lived cells in the CNS that are a source of HIV replication.

He noted that the answers to the questions as to which cell types may harbor HIV in the CNS and the nature of the cell turnover and persistence of these cellular reservoirs are not known definitively at this time.

Dr Deeks stated that in his opinion, the strongest evidence for the existence of a CNS HIV reservoir is the data from individuals who are clinically asymptomatic and have plasma HIV RNA <50 copies/ml, but in their CSF have detectable HIV (>50 copies/ml). He noted that several abstracts from CROI 2014 reported on this phenomenon of asymptomatic CSF escape, with rates ranging from 10–23 %. Of note, a published report by Eden et al., found that 10 % of 69 asymptomatic persons had evidence of CSF escape with a median detectable CSF HIV viral load of 121 copies/ml [IQR 54–213] (Eden et al. 2010). These patients versus those without CSF escape had higher levels of CSF neopterin. Notably, drug resistance could not be excluded as a cause of CSF escape because levels of CSF virus were too low to perform resistance genotyping (Eden et al. 2010).

More recently, in 49 HIV+ patients with HIV viral load measurements <40 copies/ml by conventional assays in both plasma and CSF, 17 % of patients had HIV detectable in their CSF using ultrasensitive, single-copy assays (median <0.3 copies/ml [IQR 0.2–2.9]). Importantly, CSF and plasma levels of HIV RNA were not correlated in paired samples and levels of CSF neopterin were significantly higher in those patients with versus without detectable CSF HIV RNA (Dahl et al. 2014b). These data provide further evidence that HIV cellular reservoirs may be present in the CNS and that low-level CSF RNA is associated with CNS inflammation (Dahl et al. 2014b).

### Does HIV replicate in the CNS during cART?

Dr Deeks noted that there is evidence for low-level, peripheral HIV replication occurring in some patients. These data come from studies undertaken in treated, virologically suppressed patients in whom the integrase inhibitor, raltegravir had been added to “intensify” their treatment regimens (Buzon et al. 2010; Hatano et al. 2013). In a proportion of these patients, an increase in the level of HIV DNA 2LTR circles within PBMCs was observed, suggesting that raltegravir had prevented unidentified low-level HIV viral replication from completing its integration into the host DNA (Buzon et al. 2010; Hatano et al. 2013).

As noted by Dr Margolis, one CSF raltegravir intensification study has been undertaken in 14 patients whereupon raltegravir did not reduce CSF HIV RNA levels, nor did it reduce markers of intrathecal immune activation (Dahl et al. 2011). However, Dr Deeks noted that studies aimed at proving that active HIV replication is occurring in the CNS by using integrase inhibitor intensification and observing levels of 2LTR circles have not been undertaken. Therefore, the field currently does not know if persistent viral replication occurs in the CNS during suppressive cART.

### Does inflammation persist in the CNS on treatment and is it caused by CNS infection?

Dr Deeks referred to studies providing evidence that in the periphery, the HIV reservoir size correlates with levels of inflammation and therefore, a priori, this could be true in the CNS (Barouch and Deeks 2014). More recently, a paper from his group has reported that there is an association between markers of T-cell activation and proliferation and HIV persistence (Cockerham et al. 2014).

With respect to the possible persistence of inflammation in the brain, Dr Deeks cited work by Garvey et al. who used [^11^C]-PK11195 positron emission tomography (PET) brain scanning in seven effectively treated HIV+ patients (Garvey et al. 2014). [^11^C]-PK11195 is a marker of a translocator protein, which is expressed by activated microglia. The authors found a significant increase in activated microglia in HIV+ versus HIV negative controls and that greater [^11^C]-PK11195 binding was associated with decreased executive function in HIV+ patients (Garvey et al. 2014). Dr Deeks also cited other work that described increased levels of activated microglia in postmortem studies of virologically suppressed patients (Anthony et al. 2005). Including the studies cited above where low levels of HIV RNA in the CSF were associated with higher levels of neopterin (Eden et al. 2010; Dahl et al. 2014b), Dr Deeks felt that these albeit indirect data indicate that there *is* virus replicating in the CNS which stimulates CNS macrophages to produce neopterin, and thereby provides further supportive data that the brain is an HIV reservoir.

### Can studies of systemic macrophage infection inform what is happening in the CNS?

Dr Deeks then turned to the question of whether macrophages serve as reservoirs of HIV. The current debate about the potential for macrophages to be a reservoir revolves around the fact that because macrophages phagocytose HIV-infected T cells, any HIV DNA found in the macrophages may simply be remnants of phagocytosis, presenting a technical difficulty. He cited a number of studies including work done by Mario Stevenson showing that DNA can be identified in alveolar macrophages of HIV+ patients on long-term treatment (unpublished).

Dr Deeks referred to the work of Dr Yukl where it has been consistently shown that there are non-CD4+ T-cell leukocytes that harbor HIV DNA and RNA, especially in the gut (Yukl et al. 2013) and that these may be macrophages. He also referred to other unpublished studies by Hsue et al. and Courier et al. that corroborate a potential role for macrophages as reservoirs for HIV in the periphery, which would favor the possibility that this may be the case in the CNS also.

## Session 2: eradication and the CNS model systems including cell and animal models

The first part of this session dealt with cell-based systems and commenced with latency in the macrophage and microglial cell by Dr Crowe followed by latency in the astrocyte by Dr Churchill. The session finished with latency and the neuron presented by Dr Spencer.

Dr Crowe addressed the important question of monocytes, microglia, and latency and, prefaced her talk by stating that she remains to be convinced that monocytes and microglia do serve as reservoirs for latent HIV infection.

Dr Crowe discussed several myeloid cell types that are present within the CNS including parenchymal microglia, perivascular and meningeal macrophages, choroid plexus macrophages, and trafficking blood monocytes (Prinz and Priller 2014). The turnover of these cells differs significantly with brain microglia persisting for years compared with a 2–3 month turnover of perivascular macrophages (Prinz and Priller 2014).

Until lately it was thought that all the above-mentioned cells were of the same myeloid origin. However, recent data from studies in mice show that microglia are likely to arise from uncommitted erythromyeloid stem cells in the yolk sac unlike the other CNS macrophages which arise from hematopoetic stem cell precursors in the fetal liver and embryonic aorta-gonad-mesonephros region and, postnatally, within the bone marrow (Prinz and Priller 2014). In humans, a similar pattern of development occurs with microglia first being detected in the spinal cord at 9 weeks, followed by a major influx at 16 weeks, and it is not until 35 weeks that a fully differentiated population of microglial cells can be discerned in the human brain (Prinz and Priller 2014).

Dr Crowe made the important observation that adult and fetal microglial cell lines have been used in different published studies, which make comparison between studies difficult, given the different distribution and biology of these cells in adults and fetuses.

Dr Crowe then turned to the important question of which cells in the brain can be productively infected with HIV. Perivascular macrophages, microglia, and astrocytes are all susceptible to infection but only the former two cell types permit productive infection (Gonzalez-Scarano and Martin-Garcia 2005). Perivascular macrophages are the main target for HIV in humans and are the chief target for SIV in non-human primates (Williams et al. 2001). In macaques, the density of SIV infection can vary in different areas of the brain, which may confound some studies depending on where brain tissue has been sampled and the number of sites biopsied. SIV DNA can be detected throughout SIV infection in macaques, but RNA is only present during acute HIV infection and during SIV encephalitis (Williams et al. 2001). In patients with HIV encephalitis, compared with appropriate controls, there is an accumulation of CD14+ CD16+ monocytes and perivascular macrophages within microglial nodules with p24Ag localization within these cells (Fischer-Smith et al. 2001).

Dr Crowe then reviewed the principles of latency in terms of their application in HIV infection, noting that a latently infected cell does not produce infectious virus, but that the cell’s state of latency must be reversible. She noted that the cell types that putatively support latent HIV infection include resting memory CD4+ T cells, monocytes, macrophages, astrocytes, and hematopoietic stem cells. While being convinced that memory CD4+ T cells support latency, Dr Crowe said that there is only limited evidence that monocytes and macrophages may serve as latent HIV reservoirs. Monocytes are short-lived cells that transform into macrophages within a few days and hence their brief longevity technically precludes their capacity to serve as long-acting reservoirs.

Dr Crowe noted that monocytes are widely perceived to serve as reservoirs for HIV because replication-competent HIV can be recovered from patients receiving effective long-term cART (McElrath et al. 1991; Sonza et al. 2001) and because they transform into macrophages. However, it has not yet been proven that monocytes harbor latent HIV infection in virologically suppressed individuals on cART and, if so, the frequency of latent infection.

As an aside, Dr Crowe added that if macrophages are cellular reservoirs for HIV then this poses a considerable challenge to inducing HIV remission and cure because of their longevity and their (and microglia’s) relative capacity to resist apoptosis.

Dr Crowe highlighted another reason supporting the concept that cells other than resting CD4 T+ cells serve as HIV reservoirs: when HIV+ patients have rebound HIV viremia upon ceasing cART, the rebounding virus is genetically distinct from that found in CD4+ reservoirs (Chun et al. 2000) suggesting that the rebounding virus may be coming from other cellular and/or tissue reservoirs. Phylogenetic analyses provide evidence that monocytes produce low levels of virus that can be identified in plasma in virologically suppressed HIV+ patients (Zhu et al. 2002). CD14+ CD16+ positive monocytes versus CD14+ CD16− monocytes preferentially harbor infectious virus and HIV DNA in patients receiving cART (Ellery et al. 2007).

Monocytes are harder to infect than macrophages, noted Dr Crowe. Although they have the same manner of cell entry and uncoating of the nuclear capsid, the synthesis of full-length cDNA is inefficient in monocytes (Sonza et al. 1996). Other factors may restrict susceptibility to HIV infection in monocytes eg miRNAs and cellular host restriction factors including APOBEC3G (apolipoprotein B mRNA-editing, enzyme-catalytic, polypeptide-like 3G), a family of proteins that play an important role in innate antiviral activity that restrict HIV replication in resting T cells (Chiu et al. 2005). Of note, CD14+ CD16+ monocytes contain an inactive form of APOBEC3G (Ellery et al. 2007).

The molecular mechanisms proposed that might permit HIV latency to occur in monocytes and macrophages were outlined by Dr Crowe who described them as complex. These include changes in chromatin, cellular restriction factors, and the lack of functional Tat.

Dr Crowe then turned to the question “Are monocytes latently infected with HIV?” She noted that supportive data come mostly from older studies. One study showed that freshly isolated monocytes from HIV+ persons harbored DNA but not RNA (Mikovits et al. 1992). Another study reported that infectious virus was produced when monocytes were co-cultured with activated T cells and that HIV DNA could be detected in 74 % of monocytes, although no data were provided on HIV RNA or other viral proteins (McElrath et al. 1991). However, Dr Crowe noted that it is not clear how pure the cell populations were in these studies, nor how robust the available PCR assays were.

Turning to the brain, Dr Crowe noted that circular 1-LTR DNA (Teo et al. 1997) and unintegrated DNA have been found in the brains of AIDS patients with HIVE. In one important Australian study, Thompson et al. using laser capture microdissection were able to demonstrate HIV DNA in microglia, perivascular macrophages, and astrocytes (Thompson et al. 2011). They used the absence of p24 antigen positivity on immunohistochemistry to denote that there was no productive infection in these brains (Thompson et al. 2011). However, Dr Crowe felt that today, we would require the absence of HIV RNA to be more confident about whether or not productive HIV infection was present.

Dr Crowe cautioned there are several issues that make interpreting the published data and undertaking research into latent HIV infection and microglia fairly difficult: definitions of latency may differ, hence results need to be carefully interpreted; many studies do not simultaneously examine HIV DNA, HIV RNA, and productive infection; there is a low frequency of HIV infection and integration in monocytes; the purity of cell populations (contamination with other cells) as well as the sensitivity of PCR techniques are important considerations; the location of brain tissue macrophages and microglia makes sampling of sites difficult and premortem biopsies frequently come from a single CNS site; the time from death to postmortem often limits RNA analyses. To add to these difficulties, Dr Crowe noted that adult human microglia from surgically removed brain tissue are scarce; fetal microglia differ from adult cells, there is no consensus on immunophenotypic markers for identification of microglia and the immunophenotype of resting and activated microglia differs. Some researchers thus choose the easier but far less biologically relevant route of using U1 pro-monocytic cell lines for studies of latency in monocytes.

In summarizing, Dr Crowe said she considered that it is still controversial whether true HIV latency exists in monocytes, macrophages, and microglia but it is likely, albeit unproven, that the majority of HIV+ cART-suppressed patients have reservoirs of infected cells that are transcriptionally silent in their bone marrow, thymus, and brain. Importantly, the molecular mechanisms regarding the establishment and maintenance of latency in monocytes, macrophages, and microglia remain unclear and that more rigorous studies are needed to elucidate these issues.

Dr Spencer was tasked with discussing HIV latency in the brain and therein whether CNS latency has an impact on neuronal function.

Dr Spencer began by providing a current overview of HIV treatment in middle- and high-income countries where people live with virologically controlled, chronic HIV infection. He noted that in older HIV+ populations HAND and neurodegenerative disorders are more frequently observed. At post mortem, these patient populations may have higher levels of protein misfolding and aggregation in the CNS with an accumulation of A-β protein, α-synuclein, and p-tau. The levels of A-β protein, p-tau, and α-synuclein are higher in older HIV+ patients, similar to those seen in HIV negative patients with Alzheimer’s disease and Parkinson’s disease, respectively. In gp120 transgenic mice, high levels of A-β protein and p-tau are seen suggesting that the HIV genome in the brain is associated with neurodegenerative defects that occur in older populations (Patrick et al. 2011).

Dr Spencer stated that the HIV genome in the brain takes either the form of low-level replication producing the neurotoxins Tat, nef, and gag, or the genome is fully silenced (latent). He discussed the epigenetic silencing of genes through reversible mechanisms of methylation, deacetylation, and K-9 methylation. In chronic HIV encephalitis (HIVE), viral genomic expression occurs in microglia and astrocytes via binding of Sp1 protein to the promoter region. This, in turn drives HIV genome expression through the p300 protein, via the NUCC1 promoter. By contrast, in the latent state, Sp1 binds the adaptor protein BCL11b. This promotes DNA methylation via a number of mechanisms resulting in histone modification conferring a latent state with no genomic expression.

Dr Spencer described his group’s recently published clinico-pathological study of 32 HIV+ patients whose brains were classified at postmortem according to the levels of HIV DNA, RNA and p24 antigen present (Desplats et al. 2013). Twelve patients had no DNA, RNA, or p24 antigen detected and were classified as controls. None of the controls had neuropathological findings and of those evaluable, 56 % had neurocognitive impairment (NCI). Ten patients were classified as having latent HIV infection with high levels of DNA but no HIV RNA or p24 present. A proportion of patients classified as latent had astrogliosis and 75 % of those evaluable had NCI. Finally, 10 patients were classified as being HIVE cases because they had high levels of DNA, RNA and p24 antigen. The HIVE cases had classical histopathological changes of HIVE and severe NCI was seen in this patient group (Desplats et al. 2013). Immunocytochemical and immunoblot analyses showed that, compared to those classified as controls, patients classified as either latent or HIVE had reduced synaptic neuronal markers (MAP2 and synaptophysin), increased levels of inflammation based on astroglial (GFAP) and microglial (Iba-1) markers and decreased levels of the neuronal autophagy marker, LC-3 (Desplats et al. 2013).

Interestingly, patients classified as latent had higher levels of BCL11b present in neurons and microglial cells compared to controls or HIVE patients. As a corollary, levels of BCL11b were higher in the CSF of controls and latent patients versus HIVE patients, with CSF p24 Ag detected only in HIVE patients. Furthermore, latent patients had significantly higher levels of the other chromatin modifiers HP1γ, MeCP2, and HDAC1, involved in silencing genomic expression. Finally, the authors looked for expression of other factors that are transcriptionally regulated by BCL11B. They found that there was dysregulation of expression in the patients’ brains of a number of inflammatory markers including IL-6 and TNF-α.

Using these findings, the authors have developed the following hypothesis. They reason that high levels of BCL11b present in HIV-infected microglia and astrocytes that are required to suppress active HIV infection in these cells, lead to the cells producing increased levels of pro-inflammatory cytokines such as IL-6 and TNF-α. In turn, these cytokines affect nearby neurons which, through MAPkinase signalling (Zhang et al. 2012) leads to sumoylation (or the stabilization) and accumulation of BCL11B within neurons; in turn, this leads to neuronal dysregulation. Ultimately, this would contribute to the increased levels of A-β protein, and α-synuclein that they observed also in this patient group. It also potentially explains the high levels of BCL11B that they observed in the neurons of these patients, which cannot be due to suppression of latent neuronal HIV DNA, as neurons do not support HIV infection.

Finally, Dr Spencer described their current efforts to develop an in vitro cellular model of latency to assess BCL11B impact on microglial and neuronal cells and how to reactivate latently infected cells in the brain.

Dr. Churchill started her presentation by noting that astrocytes are neuroglial cells that arise from the ectoderm, astrocytes are the most abundant cells in the brain and are responsible for maintaining brain homeostasis. Astrocytes have broad ranging regulatory effects including influences on neurotrophic factors to promote neuronal survival and myelination, on the extracellular matrix to promote synaptogenesis and neurogenesis, angiogenic factors, and endothelial cells to regulate the blood brain barrier, regulation of brain glycogen energy reserves, extracellular ions in the parenchyma, and neurotransmitter regulation. Many of these regulatory functions are disrupted in HIV infection. Astrocytes lack the usual entry mechanism for HIV, CD4 receptors, so the entry of HIV into astrocytes is CD4 receptor independent. Several potential entry mechanisms were reviewed including the mannose receptor (Liu et al. 2004), direct entry by cell to cell contact (Nath et al. 1995), CCR5 DC SIGN-dependent endocytosis (Deiva et al. 2006), endocytic uptake including CD81-dependent vesicle formation (Clarke et al. 2006; Gray et al. 2014). Prior studies have documented HIV DNA by PCR/in situ and laser capture microdissection (Takahashi et al. 1996; Sharer et al. 1996; An et al. 1999; Churchill et al. 2006). Astrocyte-specific HIV sequences have been demonstrated suggesting unique perhaps compartmentalized infection in these cells compared to other cell hosts (Thompson et al. 2004; Churchill et al. 2009). Increasing HIV DNA in astrocytes is correlated with HIV-associated dementia, an important clinical finding (Churchill et al. 2009). Interestingly, infection with HIV was found to be correlated with proximity to macrophages, and to endothelial cells (Churchill et al. 2009). Astrocytes support latent infection which appears independent of treatment with ART. HIV+ astrocytes have been documented in neurologically asymptomatic patients, and are correlated with decreasing CD4 cell counts. Dr Churchill noted the available indirect evidence for ongoing replication of HIV in the CNS during cART, citing data on biomarkers such as neurofilament light (NFL) and s100B are still increased in patients on cART over normal controls. Dr Churchill presented interesting data on the potential neurotoxicity of several compounds to reactivate latent HIV infection. She reviewed data from her lab on the relative toxicity in cell culture assays for panobinostat, romidepsin, vorinostat, HMBA, disulfarim, JQ-1, and chaetocin. These agents are of use in reactivation of latent HIV, but little is known about their ability to reactivate latent virus in astrocytes. Dr Churchill noted the differential penetration of antiretrovirals into the CNS, and went on to present data on the relative efficacy of the antiretrovirals abacavir, lamivudine, zidovudine, stavudine, efavirenz, etravirine, nevaripine, and raltegravir in reducing HIV replication in astrocytes compared to monocyte-derived macrophages and peripheral blood mononuclear cells. She found that there was reduced efficacy of lamivudine, stavudine, and zidovudine in astrocytes relative to macrophages and PBMCs (Gray et al. 2013). She noted that astrocytes were an important target in latent infection, and many questions remain regarding latent HIV in astrocytes and more research needs be done in this area.

The second half of the second session was devoted to animal models with two presentations, one from Dr J Victor Garcia on the humanized mouse model, followed by Dr Mankowski with the SIV macaque model.

Dr J Victor Garcia presented on his work on the BLT mouse model (Wege et al. 2008). He noted that HIV-1 has been associated with severe neurological issues, and that even with advanced cART, neurological functioning is still compromised although symptoms are milder. He reviewed the Trojan horse model of HIV entry into the CNS, through trafficking of HIV-infected T cells. He noted that there are many questions to be resolved about HIV CNS infection including the exact mechanism of how HIV enters the brain, what cell types are involved, the kinetics of CNS infection, whether astrocytes and microglia are latently infected cells and indeed what cells are infected. He noted that these questions cannot be answered in humans, as sampling of the CNS through brain biopsy is not feasible. Therefore, an animal model with the necessary characteristic to test the hypotheses is needed. Animal models have the ability to provide samples of all types from multiple compartments, where there is complete rigorous scientific control of the system. While non-human primate models are available, they have limitations of expense and are not human. Dr Garcia reviewed the strengths and limitations of the existing mouse models including SCID-HIVE, huPBMC, huPBL/HIVE, and huNSG (Honeycutt et al. 2014). Dr Garcia then presented the humanized BLT mouse model, created by implanting human thymus and liver tissues under the kidney capsule of immunodeficient mice. These animals are also given an autologous stem cell transplant from those same liver tissues, resulting in robust and systemic human immune cell reconstitution in these animals which are readily infectable with HIV via multiple routes. He has characterized the humanized BLT mouse brain to demonstrate that it recapitulates key characteristics of the human brain. The model has human hematopoietic cell populations, CD4+, CD8+, B, myeloid cells, dendritic cells, and CD68+ (macrophages) in relatively appropriate concentrations for the relevant research aims. In his experimental design, R5 tropic viruses are utilized in the exposure routes of vaginal, rectal, oral, and intravenous. Persistent HIV infection in the brains of the BLT mice has been shown. He noted that CD8+ cell subset relatively expanded in the CNS while the proportion of CD4+ cells decreased, and the shift occurred rapidly after infection. Antiretroviral treatment with four different regimens in the BLT mice model has been studied including TDF/FTC/DTG, RAL/TDF/FTC, RPV/DTG/FTC, and RPV/DTG with daily administration for 6–10 weeks. Dramatic reductions in both plasma and brain HIV RNA were demonstrated with ART, and the relative percentages of CD4+, CD8+, and the ratio are reconstituted and similar to uninfected mice. The BLT mice have been shown to be an extremely useful tool in investigating NeuroAIDS.

Dr. Mankowski presented on the SIV macaque model of HIV CNS latency. He opened his talk by noting that the Mississippi baby who was thought to be cured was no longer in remission. He cited Dr. Fauci’s comment on how recalcitrant the virus is and noted that we have not identified all the cellular reservoirs for HIV. He reviewed the early characterization of HIV as a member of the lentivirus family, that HIV establishes latency in macrophages, and the role of macrophages in latency. He noted that the natural hosts of SIV are in multiple African primate species such as the chimpanzee, whereas the Asian macaque is an abnormal host. The pigtailed macaque is the model of HAND that has demonstrated that SIV viral loads in the CSF are higher in those animals that develop encephalitis, compared to lower CSF viral loads in those without encephalitis. cART is effective in this SIV macaque model, with significant plasma and CSF viral RNA decay after treatment initiation. While the viral RNA reservoirs are reduced with treatment in this model, the viral DNA reservoir is not reduced. Biomarkers clinically relevant in HAND, including CSF NFL, CSF neopterin and plasma CD163 are similarly elevated in the SIV model. Dr Mankowski presented data that CNS penetrant ART reduced viral load in plasma and CSF in the SIV model, and that associated CSF inflammatory biomarkers such as MCP-1 and IL-6 were also reduced. Data were presented that demonstrated the greatly increased virulence and morbidity of SIV in pigtail macaques relative to rhesus macaques, including the development of SIV CNS disease. CCR5 inhibitors such as maraviroc (MVC) in HAND are potentially very beneficial given their high CNS penetrance, low neurotoxicity, ability to control viremia, and decrease immune activation. A study of MVC in SIV was presented with controls, where SIV in CSF and plasma was reduced by MVC monotherapy. Of interest, in addition to greatly reducing SIV RNA in the brain, SIV DNA levels in the brain reflective of latent SIV were also significantly lowered by MVC. Other potential beneficial outcomes of MVC treatment in the brain included decreased macrophage activation, lower APP accumulation, reduced CNS inflammation with both TNFα and CCL2 RNA expression lower in the brain with MVC treatment. Dr Mankowski summarized that the addition of CCR5 inhibitors including MVC to cART may prevent and treat HAND and also may reduce persistent CNS reservoirs of HIV (Kelly et al. 2013).

## Session 3: the brain as a reservoir and sanctuary

This session opened with Dr Gelman discussing the neuropathology in virally suppressed patients followed by Tat detection and expression in virally suppressed patients by Dr Johnson.

Dr Gelman began with data comparing HIV DNA versus HIV RNA in brains from well-characterised patients. He referenced his recent publications (Gelman et al. 2012a; 2013) where the point is made that latent HIV DNA in the central nervous system is not related to HAND as opposed to HIV RNA. He went further to make the point that HIV DNA distribution in the brain is not identical to HIV RNA and that the frontal neocortex has the highest burden of HIV DNA. He then showed data relating to macrophage markers with high versus low integrated HIV DNA. The macrophage markers that were associated with a high integrated HIV DNA were IRF4, CCL2 IL10, and CLEC4A. Macrophage markers that were not more common with high levels of HIV integrated DNA were AIF1, S100A9, CD16, CD14, CD163, and CD68. There were further data on IRF4 and how it is involved in macrophage polarization into the macrophage inflammatory phenotype, namely M1, versus the angiogenic wound healing phenotype, namely M2. Data were then presented localizing IRF4 to leptomeningeal macrophages. Dr Gelman then focused on evidence that the neuropathology of HAND in virally suppressed patients was a neurochemical disorder without real evidence of neurodegeneration. These data were derived from the PLoS One publication (Gelman et al. 2012a) and showed that GABAergic transmission in the prefrontal area was low, seemingly related to GAD67 downregulation in the absence of any encephalitis. Similarly, the prefrontal dopaminergic system was also abnormal with downregulation of DRD2L messenger RNA in virally suppressed patients. Indeed, low dopamine receptor 2 expression was found, even in patients who were neuropsychologically normal (Gelman et al. 2012b).

He then summarized the data for neurotransmitter abnormalities, focusing on DRD2L and enkephalin and the GABAergic system, particularly GAD1. Of these, only the enkephalin system was associated with HIV encephalitis while no system was more broadly associated with HAND. These systems were all abnormally low in the context of HIV infection per se.

Dr Gelman therefore concluded that integrated HIV DNA in the brain tissue was linked to the expression of selected macrophage products but not pan macrophage markers and that the neuropathology of HAND in virally suppressed patients was more a neurochemical disorder rather than one of neurodegeneration.

Dr Johnson then reviewed the data relating to HIV and chronic inflammation and its consequences including cognitive decline. The potential causes for chronic inflammation and T-cell activation were presented with evidence pointing to a key role for Tat. It was emphasized that current antiretroviral drugs do not inhibit Tat production as they have no effect on post integration transcription of viral components such as Tat. It was further emphasized that Tat is able to exit the cell and alter the cellular function of neighboring cells that are uninfected. The recent article by Johnson et al. (2013) was noted with in vitro evidence of darunavir’s inability to inhibit Tat production, as well as its ability to induce IL17 in T cells, leading to the hypothesis that Tat could activate T cells and produce pro-inflammatory cytokines that contribute to both inflammation and neuronal disorders.

Dr Johnson then focused on the immune reconstitution inflammatory syndrome and presented cases that were detailed in her recent publication (Johnson et al. 2013). These demonstrated CNS IRIS with no evidence of productive HIV infection but with evidence of Tat on cerebral biopsy.

As a consequence of these findings, the study was extended to determine if Tat could be playing a role in the development of HAND. A Tat ELISA that was functional in the cerebrospinal fluid was developed. Dr Johnson noted that the assay was robust with an intra-plate variability of 4.446 % and inter-plate variability of 7.56 %. The assay itself however has not been optimized for blood and is, at present, appropriate for use in only some HIV clades. Two cohorts of patients were discussed. The first comprised eight patients where three had Tat detectable in the cerebrospinal fluid. The second cohort was that of 57 patients from the North East America Dementia Cohort Study. Tat was detected in 42 % of these with some relationship to HAND severity.

In the discussion of the presentation, comment was made that a third cohort from Sydney also had shown detectable levels of Tat in the cerebrospinal fluid, but at a much lower rate. Further commentary focused on whether Tat could cross the blood brain barrier and the significance of the amounts of Tat that were present in the cerebrospinal fluid: Tat may enter the cerebrospinal fluid from HIV-infected cells in the cerebrospinal compartment, brain parenchyma, or directly across the blood-cerebrospinal fluid barrier via the choroid plexus.

## Session 4: oral abstract presentations

This session was driven by three abstracts that had been selected for oral presentation.

Dr Churchill described data relating to HIV-1 LTRs isolated from the CNS having mutations in the core promoter motif (Sp1 sites). These resulted in a reduced capacity to bind Sp1 protein and reduced transcriptional activity. It was emphasized that such unique regulatory mechanisms in the CNS will need to be considered with current HIV cure strategies as they involve activation of HIV-1 transcription.

Dr Gray showed data that HIV-1 establishes transcriptional latency in astrocytes in vitro and is responsive to the histone deacetylase inhibitors romidepsin, and Panobinostat, as well as JQ-1 while vorinostat, HMBA, disulfiram, and chaetocin showed minimal LTR activation. With the exception of chaetocin, none of these showed any significant toxicity. As such, these drugs could be considered in HIV eradication strategies.

Dr Cysique presented data on the relationship between the HIV DNA reservoir in peripheral blood mononuclear cells and HAND. In contradistinction to HAD, the more severe form of HAND, there was no relationship between non-demented HAND and HIV DNA in peripheral blood mononuclear cells. These data suggest that non-demented HAND is more related to intrinsic brain HIV disease rather than systemic HIV disease. This has direct impact on the approach to be taken for eradication strategies.

## Session 5: CNS eradication—risks and outcomes

This session was comprised of two presentations, one from Dr Robertson on initial clinical outcomes, and the second by Dr Brew on barriers and risk identification.

Dr Robertson presented on initial clinical outcomes associated with the kick and kill strategy for curing HIV. This strategy seeks to reactivate latent HIV then kills the activated and replicating virus with antiretrovirals. HIV gains entry into the CNS very early in infection. Studies during acute infection have demonstrated HIV RNA in CSF and plasma within days (Valcour et al. 2012; Spudich et al. 2011). Relative to plasma HIV RNA, CSF HIV RNA concentrations are generally lower but consistently present. Prior work by Schnell et al. (2010) provided evidence for different genetic sequences of HIV in the CNS compared to plasma, or compartmentalization of HIV. This compartmentalization was associated with HIV associated neurological disorder. Localized replication and compartmentalization has also recently been documented in primary HIV infection, as early as 4 months post infection, by the Swanstrom lab in collaboration with Spudich and colleagues (Sturdevant et al. 2014). Dr Robertson noted that if the virus is compartmentalized in the CNS, then antiretrovirals will have to penetrate the CNS to treat HAND. Letendre has defined and updated a CNS penetration efficacy ranking commonly in use for ART (Letendre 2011). It will also be necessary for eradication agents to penetrate the CNS to be effective in compartmentalized virus. While CNS penetration is necessary for eradication and treatment, there is potentially a double-edged sword to this penetration into the CNS. There is evidence that some ART can be neurotoxic as shown in vitro (Robertson et al. 2012a). While controversial, some clinical studies have found that neurocognitive performance improved with cessation of ART in an early treatment adoption group (Robertson et al. 2012b), that CNS-penetrating regimens did not improve neurocognition in those with impairment (Marra et al. 2009), a large observational study found penetrating ART associated with HAD (Caniglia et al. 2014). As ART may be neurotoxic, there should be concern that eradication agents used in reactivating the virus may also be neurotoxic. Little is known about the neurotoxicity of these agents, although some of the first data available regarding relative toxicities of these agents was presented by Churchill et al. at this meeting.

Histone deacetylase inhibitors are a potentially effective class of drugs for the kick and kill strategy. Vorinistat is one such HDAC inhibitor, which disrupts HIV latency and upregulates HIV regulation (Archin et al. 2012a). Little is known about the CNS efficacy and neurotoxicity of vorinostat (VOR). The initial clinical study of VOR enrolled eight subjects and found that VOR was effective in disrupting latency (Archin et al. 2012b). In the follow-up study with repeated dosing, a lack of induction of HIV viral replication by VOR was noted over time. Dr Robertson reported the first study of the CNS outcomes in VOR for reactivation of latent HIV, to assess initial safety and efficacy. Five subjects were assessed with a neurocognitive battery pre VOR administration and at 4 months, post 22 doses of VOR. The neurocognitive battery assessed premorbid/language (WRAT-4 Reading), verbal learning (HVLT-R), verbal memory (HVLT-R delay), fine motor (Grooved Pegboard), speed of processing (Stroop Color, Stroop Word, Trailmaking A, Digit Symbol), Attention/Working Memory (Symbol Search), and executive functions (Stroop Color-Word, Verbal fluency, Trailmaking B). There was no evidence of decline from pre and post VOR administration in global neurocognitive functioning or in specific domains of functioning, indicating very preliminary safety in this small sample. Dr Robertson noted that in future studies of HAND, eradication and cure of HIV will need to address CNS penetration, CNS efficacy, and neurotoxicity. Future studies will need to include neurocognitive and neurological assessments to assess safety and efficacy of these agents in the eradication and cure of HIV.

Dr Brew discussed the barriers to HIV eradication when HIV is present in the brain. These included the limited CNS efficacy of cART in relation to the penetration of ARVs into the brain, the limited and variable cellular efficacy especially in astrocytes (Gray et al. 2013) and the inefficacy in preventing post integration transcription so that potentially neurotoxic viral components such as tat could still be produced as previously discussed (Johnson et al. 2013). Moreover, currently there are no validated tools by which to quantitate the amount of latent HIV in the brain. Biomarkers such as magnetic resonance spectroscopy and BCL11b (Desplats et al. 2013) require more extensive rigorous study. For example, does a normal magnetic resonance spectroscopy mean there are no significant amounts of HIV DNA in the brain? The risks of HIV eradication relate to worsening of CNS viral burden because current ARVs cannot completely control brain HIV infection. This is pertinent to the “kick and kill” strategy where there is “reawakening” of HIV from its latent state and then killing, as well as the functional cure strategy. In both scenarios, an IRIS phenomenon could develop, analogous to that which occurred in Alzheimer’s disease with active immunization against amyloid (Brew et al. 2013). Further risks were discussed including the toxicity of therapies, both ARVs and HDAC inhibitor drugs as well as the potential for off-target effects especially modulation of gene activity. The solutions to these issues have yet to be found but given that at most approximately half of HIV-infected patients do not have HAND it would seem reasonable to focus on such patients for eradication therapies. Further, it may even be possible to include patients with little or no HIV brain disease by targeting those at seroconversion or at least early in the course of HIV disease. The development of better cART regimens that have good CNS penetration and block entry into cells as well as inhibiting post integration transcription will allow the inclusion of at least some HAND patients. The critical issue will be the quantification of the HIV brain reservoir—those HAND patients with a low burden may be able to tolerate mild IRIS. There may be situations where mild neuronal loss will be an acceptable “price” for HIV eradication, similar to the “chemo brain” that some patients experience as a consequence of systemic chemotherapy as treatment for a variety of cancers.

## Session 6: current and future studies

Dr Jeymohan Joseph in this session dealt with NIMH priorities and funding for HIV eradication with the focus on the brain along with contributions from Drs Dianne Rausch and Deborah Colosi. Dr Joseph stated that the Division of AIDS Research at NIMH was keen to support research directed goals to:

- Identify and characterize persistent HIV-1 in CNS-derived cells in the setting of suppressive cART,
- Determine the mechanisms involved in the temporal establishment, maintenance, and resurgence of persistent HIV-1 in the CNS in relationship to the timing of cART
- Develop physiologically relevant animal models and CNS-based cellular assays that recapitulate HIV-1 persistence and latency in the presence of effective cART
- Assess current and emerging eradication approaches to determine if they have successfully reactivated persistent HIV from CNS-derived cells
- Assess CNS toxicity and any adverse impact of current and emerging eradication strategies

The strategies to achieve these goals included organizing HIV CNS eradication focused brainstorming workshops, issuance of funding opportunities announcements (FOAs), and provision of resources for HIV CNS eradication research. NIMH has partnered with other sister institutes such as NIAID, NINDS, and NIDA in the issuance of FOAs targeted at stimulating research on HIV persistence in the brain. Dr Joseph also described the availability of tissue resources for CNS Eradication research from the National NeuroAIDS Tissue Consortium (NNTC) (https://nntc.org/). The NNTC, supported by NIMH and NINDS, had four Clinical Sites in the US and can provide brain, CSF, and other tissue samples to qualified investigators.

## Conclusions

The symposium concluded that the evidence for the brain as a significant HIV reservoir, while compelling, still requires further substantiation with attention to controversial issues such as latent infection of intrinsic brain cells, and that the brain may be a significant HIV reservoir only in some patients. Further research also needs to focus on the development of biomarkers to identify and quantify the HIV reservoir in the brain. Finally, the brain should be a primary concern in the development of new eradication strategies.
